# Molecular Clusters Size of *Puerariae thomsonii* Radix Aqueous Decoction and Relevance to Oral Absorption

**DOI:** 10.3390/molecules200712376

**Published:** 2015-07-07

**Authors:** Gong Wang, Caimei Yang, Kuan Zhang, Juan Hu, Wensheng Pang

**Affiliations:** 1 The Institute of Drug Research, Fujian Academy of Traditional Chinese Medicine, Fuzhou 350003, China; E-Mail: hujuanketizu_wg@163.com; 2 The College of Pharmacy, Fujian University of Traditional Chinese Medicine, Fuzhou 350122, China; E-Mails: ycm0408@126.com (C.Y.); hujuanketizu_zk@163.com (K.Z.); 3 The Second People’s Hospital of Fujian Province, Fuzhou 350003, China; 4 Zhangzhou Health Vocational College, Zhangzhou 363000, China

**Keywords:** *Pueraria thomsonii* Benth, water decoction, molecular aggregates, HPLC fingerprint, absorption behavior

## Abstract

The multi-component system of traditional Chinese medicine (TCM) is very complicated. The clusters are dynamic aggregates whose molecules are held together by hydrogen-bonded, Van der Waals forces or the opposite charges of particles attract each other. In this paper, field emission scanning electron microscopy proved that molecules form clusters in *Pueraria thomsonii* Benth (Fenge) water decoction. Four kinds of Fenge water decoction, 0.07 g∙mL^−1^ (F-1), 0.1 g∙mL^−1^ (F-2), 0.17 g∙mL^−1^ (F-3), 0.35 g∙mL^−1^ (F-4); F-1, average diameter of molecular was about 120 nm; F-2, 195 nm; F-3, 256 nm; and F-4, 480 nm. The molecular size was shown to depend on concentration. Rabbits were given equal does of 2.8 g∙kg^−1^, to perfuse F-1, F-2, F-3, F-4 in volume of 80 mL, 56 mL, 33 mL, 17 mL, respectively. At 0–180 min to collect 2 mL blood from the rabbit ears middle arteries for metabolism fingerprints, the results show the particle size of molecular is smaller, the absorption of drugs is better instead. The acute blood stasis model rats were treatment with Fenge decoction of 1.5 g∙kg^−1^ for 14 days, the concentrations of Ang II in plasma were significantly lower in F-1 and F-2 groups than those in model group (*p* < 0.01 or *p* < 0.05), but there were no significantly difference in F-3 and F-4 groups than those in model group (*p* > 0.05). Despite the molecular aggregation is a common physical phenomenon, it influence on the kind and amount of molecule per unit volume. Molecules morphology influence on the absorption behavior of drugs *in vivo* therefore is to have an impact on pharmacological function.

## 1. Introduction

The number of drugs observed to aggregate or to form micelles in aqueous solution continues to increase. This is no entirely a surprising observation because the structural requirements for surface activity or micelle formation are often similar to those for interaction of drug with receptor sites. Cooperative aggregation phenomenon for drugs is quite similar to that of surfactants; they aggregate at concentrations above a threshold value, called the “cmc”. The threshold concentration is about 10^–2^ mol∙L^−1^, although premicellar aggregates containing two or more drug monomers have also been described [1,2].

It is known that lipophilic compounds (such as TMC120 and R278474) can form aggregates [3]. Formation of aggregates is common, and it may be the cause of nonspecific binding to proteins that has been observed in high-throughput screening (HTS) tests.

The small hydrophobic drug molecules such as Diarylpyrimidine (DAPY), Non-nucleoside reverse transcriptase inhibitors (NNRTIs) that bind to a hydrophobic pocket of the HIV-1 reverse transcriptase (RT). It has exceptional inhibitory properties against drug-resistant to viral strains of HIV-1 and good oral bioavailability. Assume that inhibition of RT results from endocytosis of a single aggregate of inhibitor molecules by the infected cell. Knowing the EC_90_ of the DAPY compounds (about 5 times the EC_50_) and the concentration of infected cells in the test medium (150,000 cells/mL), it is possible to estimate the number of inhibitor molecules per aggregate as approximately 2,000,000. Taking into account the average volume of a DAPY molecule (0.38 nm^3^) yielded an estimate of 57 nm for the theoretical radius of an aggregate [4]. Under various conditions mimicking physiological transitions in the GI environment, aggregate size distributions were shown to depend on compound concentration and pH [5].

Drug aggregates may be absorbed via the transcellular pathway through epithelial cells (enterocytes) that line the intestinal wall or through specialized microvilli (M-) cells that cover lymphoid follicles (Peyer’s patches) [6,7]. Highly fat-soluble substances (such a vitamins A and D) are known to be well-absorbed [8] and transported via the lymphatic system [9]. The gastrointestinal system is extensively permeated by a lymphatic network that drains via the cisterna chyli into the thoracic duct, which in turn unites with the blood circulatory system at the junction of the left internal jugular and subclavian veins [10]. These absorption mechanisms may explain the good oral bioavailability of some highly lipophilic and poorly water-soluble compounds.

Using Particle Size Analyzers, dynamic light scattering (DLS) and electron microscopy (EM), formation of drug aggregates was demonstrated. DLS measures the normalized (second-order) time autocorrelation function of scattered-light intensity. For Brownian particles, this quantity is related to the normalized (first-order) autocorrelation function by the Siegert relationship. For themorphology studies, freeze-dried samples were visualized using a scanning electron microscope. Samples were dusted on a double-sided adhesive tape previously applied on a stainless steel stub. All samples were then sputter-coated with gold prior to microscopy examination [11]. Malvin NanoSight visualizes measures and characterizes virtually all nanoparticles. Particle size, concentration, aggregation and zeta potential can all be analyzed while a fluorescence mode provides speciation of labeled particles. NanoSight provides real time monitoring of the subtle changes in the characteristics of particle populations with all of these analyses uniquely confirmed by visual validation.

Some molecules can form aggregates in solution that may inhibit several enzymes, known as promiscuous inhibitors. This phenomenon has been found in traditional Chinese medicinal recipes [12]. Previous works of authors’ research group demonstrated that aggregates are commonly existed in water decoction of Traditional Chinese Medicine (TCM) by means of DLS and TEM [13]. The efficacy of Chinese herbal formulae against multiple targets was aggregates-related [14]. Exceptional bioavailability properties of TCM were hypothesized to come from their ability to form the congeries of different molecules held together by chemical forces, we shall perhaps explain the efficient substance of multi-constituents cooperate with each other which are from the gastrointestinal tract uptake pathway into systemic circulation.

*Pueraria thomsonii* Benth a variety of Radix Puerariae (sometimes called Fenge). is rich in isoflavonoids, and has been used to treat angina pectoris, hypertension, influenza, and neck stiffness, *et al.* Puerarin is the main active component Radix Puerariae, also has various pharmacological activities, including anti-hypertensive, hypolipidemic, anti-diabetic, and cardioprotective [15]. Puerarin has an antihypertensive effect, and its mechanism may be related to inducing the changes of apelin-12, angiotensin II and NO, and regulating the balance among those factors [16,17]. Fenge also contain much starch, the associations are more likely to occur when liquids whose molecules are held together by hydrogen bonds, Van de Waals forces and hydrophobic forces. However, the effect of different concentrations of Fenge water decoction on curative effect is not well documented.

In this paper, we use field emission scanning electron microscopy (FE-SEM) and nanoparticle tracking analysis to study aggregation morphology of molecules and characterize their particle size of Fenge water decoction. To investigate the absorption characteristic of Fenge water decoction with different molecule scales in rabbit plasma by the high performance liquid chromatography fingerprint. The octapeptide angiotensin II (Ang II) is an essential contributor to vascular tone, and to fluid and electrolyte homeostasis. Different concentration of Fenge water decoction, adjust the volume of perfusion, all animals received drug of equal does. By measuring the Ang II in hypertensive rats model in order to probe into the relationship among molecular clusters size, internal absorption, and efficacy of different concentration decoction.

## 2. Results and Discussion

### 2.1. The Molecules Aggregates Characterization of Different Concentrations of Fenge Water Decoctions

**Figure 1:**
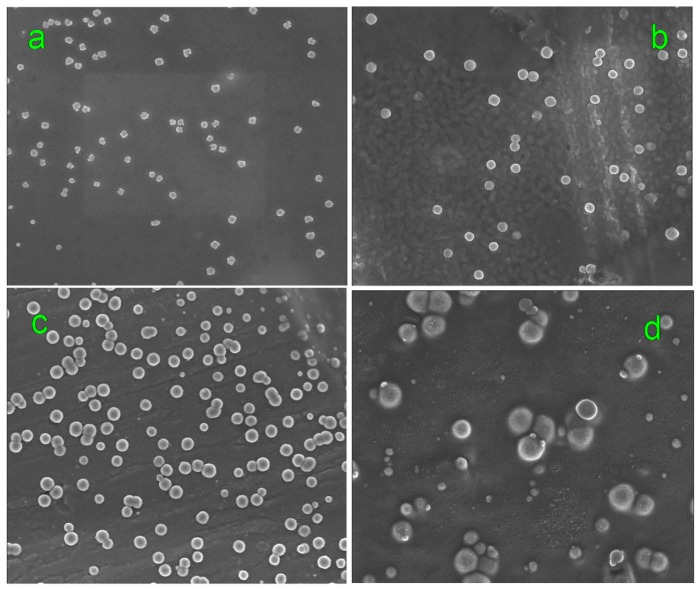
TEM images of molecules of Fenge water decoction. (**a**–**d**) display the characteristic cluster structure of F-1, F-2, F-3, and F-4.

**Figure 2:**
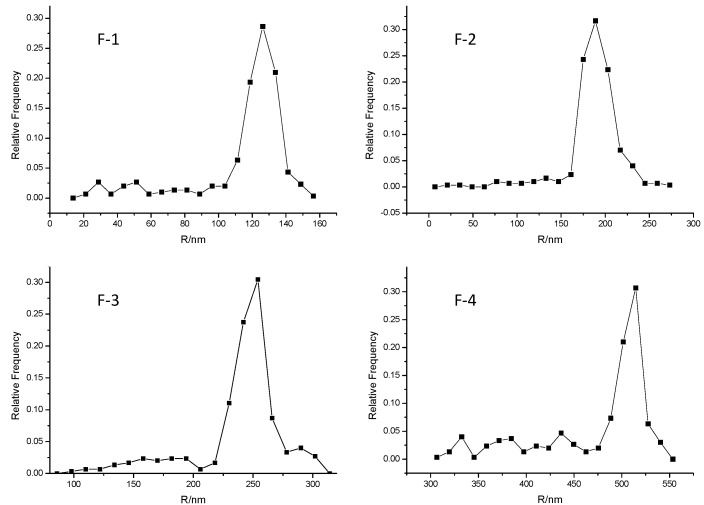
The particle size distribution graph of molecules in Fenge water decoction.

FE-SEM results showed the molecules aggregates in F-1 ranged from 110 to 135 nm in diameter and had an peak value of 120 nm; F-2 ranged from 160 to 230 nm in diameter and had an peak value of 185 nm; F-3 ranged from 220 to 260 nm in diameter and had an peak value of 245 nm; F-4 ranged from 475 to 520 nm in diameter and had an peak value of 500 nm. See in Figure 1 and Figure 2. The aggregate size increase with the concentration increases of Fenge solution and the distributions were shown to depend on solution concentration. The size (diameter) of molecule aggregates of four kinds of solution was in the following order: F-1 < F-2 < F-3 < F-4. Chinese medicine is a complicated multilevel system including multi-component, multi-targets, interactions of crossover network, *etc.* The molecular clusters may be aggregates of molecules, or mixed materials. Between aggregated molecules, there may be strong interactions of chemical bonding or nonbonding weak interactions, such as Van de Waals forces, hydrogen bonds and hydrophobic forces among multi-component molecules. So molecules can form aggregates in solution that may inhibit several enzymes, known as promiscuous inhibitors.

### 2.2. Particle Size Tracking Analysis of Fenge Water Decoction

Nanoparticle tracking analysis (NTA) is a method for visualizing and analyzing particles in liquids that relates the rate of Brownian motion to particle size. The rate of movement is related only to the viscosity and temperature of the liquid; it is not influenced by particle density or refractive index.

**Figure 3:**
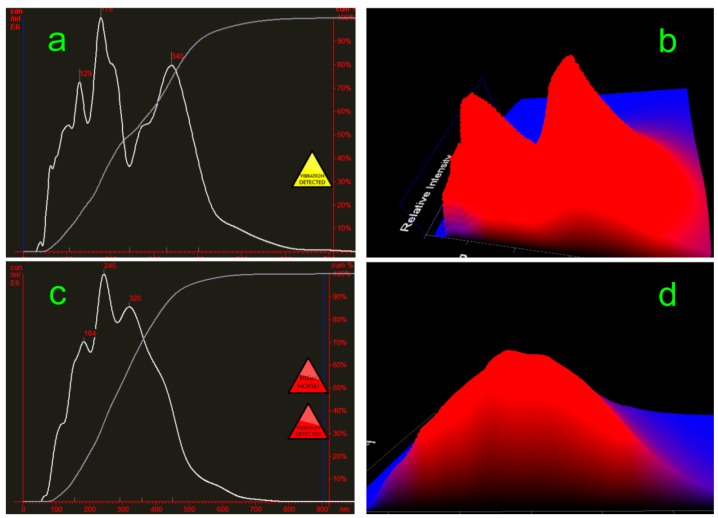
Graphs of particle size/concentration and particle size/relative intensity 3D plot of F-2 and F-3. (**a**,**b**) display the particle size distribution of F-2; while (**c**,**d**) display F-3.

NanoSight NS 300 operated at liquid volume 1.0 mL with measurement time 60 s and the measurement temperature 23 °C. Figure 3a–d display graphs of particle size/concentration and particle size/relative intensity 3D plot of F-2 and F-3. In F-2 solution, particle size was average diameter 195 nm, concentration 3.34 × 10^8^ particles/mL; F-3 solution, particle size was average diameter 256 nm, concentration 3.75 × 10^8^ particles/mL. Particle size became greater as the concentration increased. At the submicroscopic level a molecular clusters appears to be an incalculable chaos of moving molecules.

### 2.3. Fingerprints of Fenge Water Decoctions

Construction of the chromatographic fingerprints plays an important role in the multi-component separation and determination of complex nature products. Here the authors optimize the key determine parameters for HPLC chromatographic fingerprint establish of the Fenge decoction. The wave1engths were selected by a PAD full wavelength scan of 190–400 nm. Discriminating the characteristic peaks of flavonoids, daidzein had the best responses at 250 nm, especially with the retention time of 5–60 min in which 10 marker compounds showed the greatest absorbance. Therefore, the wavelength of 250 nm was chosen to detect the target components.

**Figure 4:**
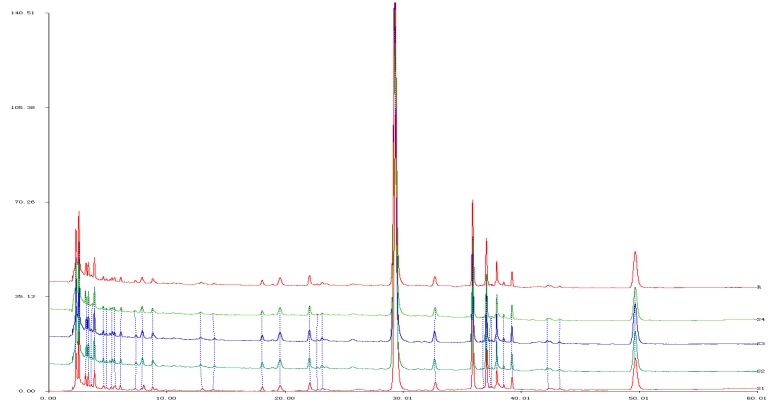
The superposed graphs of HPLC fingerprint of 4 Fenge water decoctions. The HPLC referential fingerprint from (R).

The chromatographic fingerprints were analyzed in four decoctions of Fenge using the Similarity Evaluation System of Chromatographic Fingerprint of Traditional Chinese Medicine. There were 10 co-possessing peaks and the fingerprints of all specimens showed a greater than 98% similarity. The RSD of the relative retention time as well as that of the relative peak area of the shared peak was lower than 3% in the precision, stability and reproducibility tests. The chemical components of Fenge decoctions are of good consistency see in Figure 4.

### 2.4. Fingerprints of Rabbit Plasma

Overnight fast, rabbits were oral perfuse a single dosage of 28 g∙kg^−1^ body weight of four kinds of Fenge water decoction that was different diameter of particle. At 0 min, 10 min, 30 min, 60 min, 120 min and 180 min after administration, collect 2 mL blood from the rabbit ears middle arteries gently invert the tube 3 to 5 times to allow EDTA anti-coagulant to thoroughly mix with the blood for metabolism fingerprints. To obtain baseline blood metabolism level from control group.

Allow blood to clot at room temperature for 1 h. Centrifuge the specimen at 3500 rpm∙min^−1^ for 15 min then carefully pipette the clear plasma (supernatant) to a clean specimen tube using a plastic pipette. Do not disturb the clot. Aliquot it into pre-labeled plastic, screw-cap vials and store at −20 °C. Avoid freeze-thaw cycles. Rabbit’s plasmas were pre-treated by perchloric acid before determination.

200 μL plasmas were precipitated with 50% perchloric acid in 1:10 proportions. After vortex mixing for 2 min, centrifuge the specimen at 12,000 rpm∙min^−1^ for 10 min then pipette the supernatant. The plasma samples were determined the chromatogram of pharmacochemical ingredient by HPLC. Separated was on SHIMADZU Shim-pack VP-ODS (4.6 mm × 150 mm, 5 μm). The mobile phase was acetonitrile-0.03% phosphoric acid aqueous solution. The gradient program included four linear steps, the ratio of acetonitrile was raised from 5% to 25% in first 0–15 min, and then at 30 min, and 35 min. But phosphoric acid was increased from 95% to 75% respectively, see in Table 1. The flow rate was 1.0 mL∙min^−1^ and detected at 250 nm.

**Table 1:** The gradient elution program of the HPLC.

Time (min)	A% (Acetonitrile)	B% (0.03% Phosphoric Acid)
0	5	95
15	10	90
30	14	86
35	25	75
60	25	75

**Figure 5:**
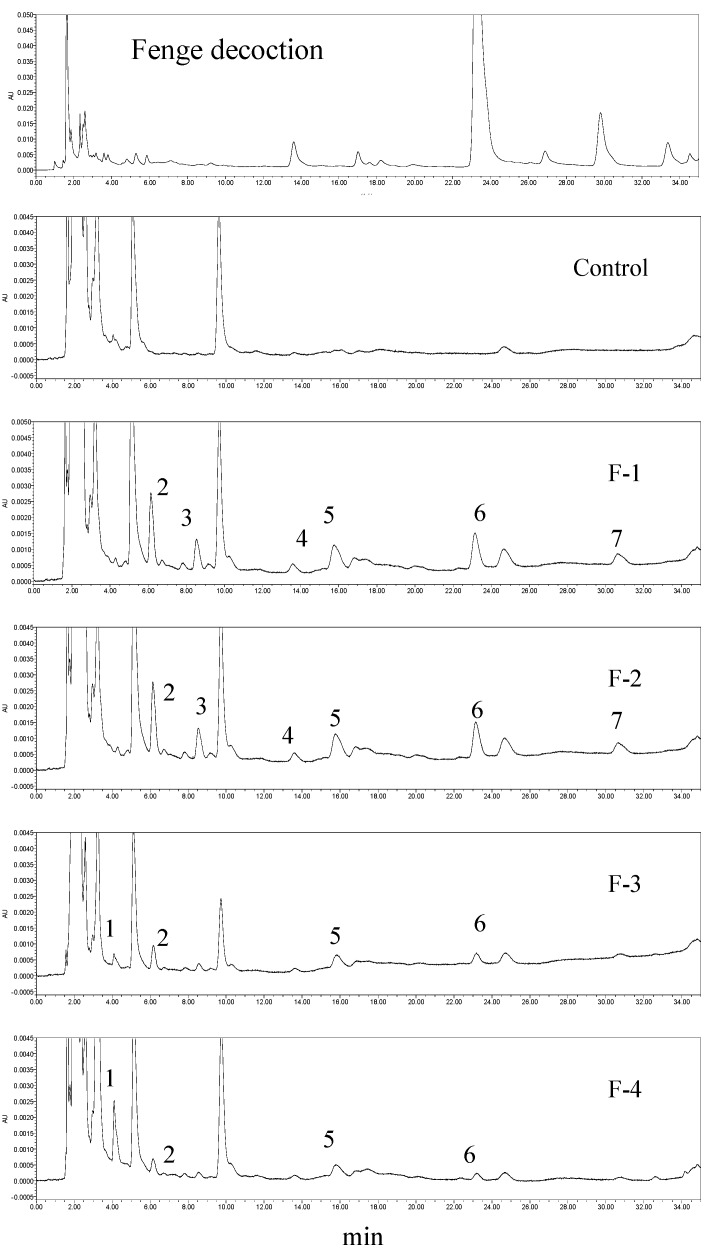
Fenge water decoction fingerprint and plasma fingerprints after rabbits were treated with different concentration of F-1, F-2, F-3, and F-4.

The peak areas of the components were calculated for every three rabbits at each time point. Compare fingerprint with different time, Fenge was absorbed and removed quickly in stomach, the plasma drug concentration had been increased as the highest achieved in 30 min. In Figure 5 showed that there were 7 chemical compositions absorbed into blood in the medicinal plasma, among them 4 peaks were original compositions (labeled as 4–7) came from Fenge decoction; 3 peaks were metabolites (labeled as 1–3). The ingredients of main peaks of chromatogram of the Fenge decoction, peaks 4–7 were absorbed into blood in a higher proportion. Peaks 1–7 retention time was 4.1, 6.2, 8.6, 13.7, 15.7, 23.1, as well as 30.7 min, respectively. Ingredient of peak 1 was absorbed and removed slowly, after rabbits were given F-1 and F-2, Peak 1showed no signs; F-4 surpassed F-3 in peak 1 area. The rest 6 peaks chemical compositions were all absorbed into systemic circulation with peak area value in the order of F-1 > F-2 > F-3 > F-4.

**Figure 6:**
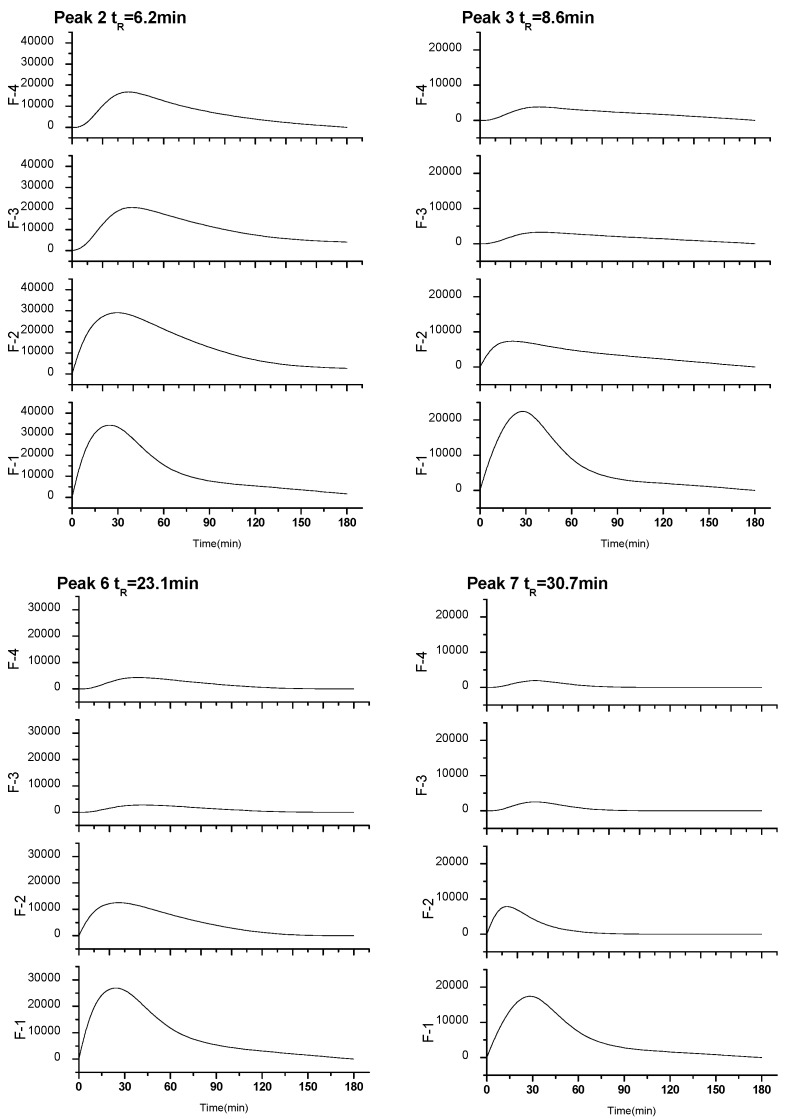
The time-dependent peak area curve of four kinds of constituents absorbed into blood after four different concentration of Fenge water decoction oral administration, ingredients of peaks 6 and 7 were original compositions, while peaks 2 and 3 were metabolites.

Figure 6 illustrated that the time-dependent peak area curve of four kinds of peak 2, peak 3, peak 6, and peak 7 ingredients absorbed into blood after four different diameter of particle of Fenge decoction oral administration, the peak time was about 0.5 h, all ingredients exhibited short mean residence time less than 2.8 h among which peak 7 showed the shortest MRT of 1.05 h. The result of experiment embodied that molecular clusters size increases with concentration of Fenge solution increasing, the absorption of drugs is not better.

### 2.5. Pharmacodynamic Assessments of Four Kinds of Fenge Decoction with Different Diameter in Rats

Angiotensin II (Ang II) is a hormone that may act on the central nervous system to regulate renal sympathetic nerve activity, renal function, and, angiotensin II involved in the regulation of blood pressure, water and sodium homeostasis, and control of other neurohumoral systems. The acute blood-stasis model rats with adrenalin, ang II level increased significantly (*p* < 0.01) compare with control group. Model animals were received four kinds of Fenge water decoction with different diameter. Positive control group was administered to Compound Salvia tablets suspension that daily dose was 0.45 g∙kg^−1^. Negative control and model control group rats were received physiological saline of equal volumes. All groups were administered for 15 days. Blood samples (2 mL) were taken from the abdominal aorta of each animal into a syringe, was immediately transferred to a chilled 5 mL polystyrene tube containing Na_2_EDTA. The pharmacodynamic effect was evaluated by monitoring the plasma Ang II level with specific radioimmunoassay as previously reported [18]. After the treatment, the concentrations of Ang II in plasma were significantly lower in group F-1 and group F-2 than those in model group (*p* < 0.01 or *p* < 0.05), could obviously ameliorate the hemorbeological parameters. But there were no significantly difference in group F-3 and group F-4 than those in model group (*p* > 0.05), see in Table 2. The formation of molecular aggregation leads to change of absorption behavior of drug therefore is to have an impact on pharmacological function.

**Table 2:** Content of Ang II in plasma in all groups ($\overset{-}{X}$ ± s).

Group	*n*	Ang II (pg∙mL^−1^)
Control	10	152.34 ± 23.20
Model	10	272.55 ± 28.72 **
Positive control	10	173.09 ± 28.78 ^ΔΔ^
F-1	10	183.50 ± 31.23 ^ΔΔ^
F-2	10	198.96 ± 38.86 ^Δ^
F-3	10	232.97 ± 31.88
F-4	10	248.21 ± 29.53

^Δ^ *p* <0.05, ^ΔΔ^ *p* < 0.01 *vs.* model group; ** *p* < 0.01 *vs.* control group.

## 3. Experimental Section

### 3.1. Fenge Medicinal Slices and Reagents

Fenge medicinal slices were purchased from Fujian University of Traditional Chinese Medicine Guo Yi Tang Hospital, Fuzhou City, China. HPLC grade methanol and acetonitrile were purchased from Merck (Darmstadt, Germany). Analytical grade reagents were purchased from Sinopharm Chemical Reagent Co. (Shanghai, China). Deionized water was purified by the Milli-Q-Plus ultra-pure water system (Milford, MA, USA).

### 3.2. Instruments

Model Nova Nano SEM 230 Field emission scanning electron microscope (Hillsboro, OR, USA) with Gatan sample heating plate (Pleasanton, CA, USA). The samples were then coated with a 2 nm layer of gold using an Emitech K550X automated sputter coater. Image acquisition and analysis system of Powersite 410 was used. NanoSight NS 300 nanoparticle tracking analysis system, NTA (Malvern, UK), Malvern instruments’ NTA application software running in a Windows environment and offering a user-friendly graphics interface for analysis and data manipulation functions. HPLC system was performed with a Waters Technologies (Milford, MA, USA) Alliance 2695 separation module equipped with an auto-sampler and a Waters 2996 photodiode-array detector (PDA).

### 3.3. Samples Preparation

Fenge medicinal slices were crushed to powder (60 smash). Weigh four different doses of 50.0 g, 70.0 g, 120.0 g and 240.0 g Fenge medicinal slices. Added in six times cold-water to soak in 30 min and let it simmer 30 min. Repeat again, after filtration and combination of two filtrates. Make the volume to around 700 mL, the concentration of four decoctions was 0.07 g∙mL^−1^ (F-1), 0.1 g∙mL^−1^ (F-2), 0.17 g∙mL^−1^ (F-3), 0.35 g∙mL^−1^ (F-4), respectively.

### 3.4. Animal Experiment

#### 3.4.1. Four Kinds of Fenge Decoction with Different Diameter in Rabbit Absorption Behavior

New Zealand rabbits were purchased from Shanghai Slac Laboratory Animal Co., Ltd. (Shanghai, China) and kept at 20–25 °C and constant humidity 45%–65% under a 12 h light-dark cycle with free access to food and water. The experimental procedures were carried out in accordance with the Guidelines for Animal Experimentation of Fujian University of Traditional Chinese Medicine (Fuzhou, China). Fenge decoctions were administered to rabbits at a does of 2.8 g∙kg^−1^, to perfuse F-1, F-2, F-3, F-4 in volume of 80 mL, 56 mL, 33 mL, 17 mL, respectively.

#### 3.4.2. Four Kinds of Fenge Decoction with Different Diameter in Hypertension Rat Treatments

Seventy male Wistar rats (250 ± 20 g) were divided randomly into seven groups, and 10 rats per group. Normal control and model group where the animals received a single oral dose the same volume saline served as the vehicle control; positive control group was intragastrically administrated with 0.45 g∙kg^−1^ Danshen tablets suspension; drug groups, rats treated with four kinds of different concentration of Fenge water decoction which was administrated at the same dose of 1.5 g∙kg^−1^. All groups were administered for 15 days.

Besides normal control group, after the last administration in rats of each group were given adrenalin hydrochloride (0.8 mg∙kg^−1^) subcutaneously twice with an interval of 4 h. During the interval between two injections, ice-cold water was used to induce acute blood stasis model. The animal studies were approved by the Fujian Academy of Traditional Chinese Medicine Animal Ethics Committee (Fuzhou, China).

### 3.5. Molecules Aggregates Size of Fenge Water Decoction

#### 3.5.1. Molecules Aggregates of Fenge FE-SEM Observation

The specimen was placed on the carbon membrane support (copper wire mesh) in a sputter coater and the excess of liquid drained at the temperature 60 °C for 10 min to stain by 2% phosphotungstic acid and finally the specimen was coated with gold/palladium at 25 mA for 10 min. The morphology of molecules was observed in FE-SEM Fei Nova Nano SEM 230 operated at accelerating voltage 7.0 kV with a working distance around 5 mm and the room temperature.

#### 3.5.2. Nanoparticle Tracking Analysis of Fenge Water Decoction by NTA

Nanoparticle Tracking Analysis (NTA) detects and visualizes populations of nanoparticles in liquids down to 10 nm, dependent on material, and measures the size of each particle from direct observations of diffusion. Additionally, measures concentration and a fluorescence mode differentiate labeled particles within complex background suspensions.

### 3.6. Chromatographic Analysis

Chromatographic separation was achieved on a SinoChrom ODS-BP (4.6 mm × 200 mm, 5 μm) column. The mobile phase was acetonitrile-0.03% phosphoric acid aqueous solution. The gradient program included five linear steps, the ratio of acetonitrile was raised from 5% to 25% in first 0–15 min, and then at 30 min, 35 min and 60 min. But phosphoric acid was increased from 95% to 75% respectively. The flow rate was 1.0 mL∙min^−1^.

### 3.7. Blood Collection for Ang II Radioimmunoassay

Blood samples (2 mL) were taken from the abdominal aorta of each animal into a syringe, was immediately transferred to a chilled 5 mL polystyrene tube containing Na_2_EDTA. The collection tube was inverted repeatedly followed by centrifugation (10 min at 3000 rpm and 4 °C) and supernatant of the plasma which was immediately frozen for storage at −20 °C. Angiotensin peptides were extracted from 1 mL plasma using phenylsilica reverse phase cartridges as previously described, prior to being resuspended in buffer to give a 2.5 fold concentration and subjected to radioimmunoassay. Briefly rabbit anti Ang II sera and ^125^I-Ang II were added to sample extracts, incubated at 4 °C for 16 h and the bound and free ^125^I-Ang II separated using a solid phase second antibody method. Results were interpolated from a standard curve.

### 3.8. Statistical Analysis

Results were expressed as mean ± standard error and all statistical comparisons were made by means of a one-way (ANOVA) test and were analyzed with SPSS, version 13.0 software. Values of *p* < 0.05 were considered to be statistically significant.

## 4. Conclusions

The chemical compositions in blood may be the drug-like compositions with the direct actions *in vivo*, which come from the ingredients original compositions or metabolites. The chromatogram of pharmacochemical ingredient of plasma of rabbits by HPLC is studied for the first time. The chromatogram can show all the changes of ingredients that are absorbed into blood after administration different concentration of Fenge water decoction with different diameters of particle. The concentration is lower, the particle size is small, the absorption of drugs is better. The dilution can’t exceed certain limits yet. Our data suggested certain strong interactions among the multi-components molecules of TCM decoction.

Though “molecular aggregation” is a common physical phenomenon, however, these systems, showing different self-assembling structures, exhibit a much richer and interesting phenomenology; it plays an important role in absorption of drugs, far from being completely understood. Our data offer references for clinical rational use of Chinese medicine. We eager new experiences and will ascertain the pharmacodynamic substance and metabolism mechanism of TCM.
